# Native‐like Env trimers as a platform for HIV‐1 vaccine design

**DOI:** 10.1111/imr.12481

**Published:** 2017-01-30

**Authors:** Rogier W. Sanders, John P. Moore

**Affiliations:** ^1^ Department of Microbiology and ImmunologyWeill Medical College of Cornell UniversityNew YorkNYUSA; ^2^ Department of Medical MicrobiologyAcademic Medical CenterUniversity of AmsterdamAmsterdamThe Netherlands

**Keywords:** Env trimers, HIV‐1 vaccines, neutralizing antibodies

## Abstract

We describe the development and potential use of various designs of recombinant HIV‐1 envelope glycoprotein trimers that mimic the structure of the virion‐associated spike, which is the target for neutralizing antibodies. The goal of trimer development programs is to induce broadly neutralizing antibodies with the potential to intervene against multiple circulating HIV‐1 strains. Among the topics we address are the designs of various constructs; how native‐like trimers can be produced and purified; the properties of such trimers in vitro and their immunogenicity in various animals; and the immunization strategies that may lead to the eventual elicitation of broadly neutralizing antibodies. In summary, native‐like trimers are a now a platform for structure‐ and immunology‐based design improvements that could eventually yield immunogens of practical value for solving the long‐standing HIV‐1 vaccine problem.

This article is part of a series of reviews covering B cells and Immunity to HIV appearing in Volume 275 of *Immunological Reviews*.

## 1 Introduction

The ultimate goal of HIV‐1 vaccine research programs is to design and develop an immunization strategy that is both protective and practical. By protective, we mean that the vaccine formulation must have a meaningful level of efficacy at preventing the acquisition of new HIV‐1 infections, irrespective of the transmission route and the geographic setting. By practical, we mean that the vaccine components must be capable of being manufactured and used in a real world environment. Various different strategies that are being pursued toward accomplishing these challenging goals will be reviewed in this volume. Here, we will discuss the design, production, and application of recombinant protein mimics of the native envelope glycoprotein (Env) spike that is present on virus particles, that mediates virus entry into target cells, and that is the target for virus‐neutralizing antibodies (NAbs) raised by the immune system of the infected host.

Most licensed vaccines confer protection against viruses by inducing antibodies that recognize and counter the relevant pathogen.^1^ Thus, an effective immunogen, whether a live‐attenuated or killed version of the pathogen or a soluble subunit protein that is usually given in combination with an adjuvant, induces an antibody response of a sufficient quantity, quality, and duration to prevent infection when the cognate virus is encountered. The specific antibody correlate of protection is not always known but usually involves NAbs, as quantified by in vitro infection inhibition assays.^1^ In general, a NAb works by binding to a functional antigen on the virus particle and preventing that particle from entering and infecting a susceptible target cell. Antibodies have other antiviral functions that are mediated by their Fc regions and generally target already infected cells. The relevance of these effector functions to HIV‐1 vaccines will be addressed in other articles in this volume. Here, our emphasis will be on immunogens designed to induce NAbs.

The extraordinary global sequence diversity of HIV‐1, and hence its extreme antigenic diversity, requires that practical approaches focus on inducing not just NAbs in general, but a subset that has a sufficient breadth of activity against multiple circulating strains.^2^, ^3^, ^4^, ^5^, ^6^ These broadly neutralizing antibodies (bNAbs) can emerge in approximately 20–30% of HIV‐1‐infected people, an observation that provides an important proof of concept for vaccine strategies.^7^, ^8^, ^9^, ^10^, ^11^ Thus, the human humoral immune system is certainly capable of producing bNAbs against HIV‐1, under the right conditions. Moreover, the individual and collective properties of bNAbs serve as critical information for the design and assessment of Env immunogens, particularly those we cover in this article.^2^, ^3^, ^4^, ^5^, ^6^ The rationale is as follows: The host immune system can sometimes respond to HIV‐1 infection by inducing bNAbs that recognize relatively conserved epitopes on the Env spike. So, can Env spikes be used as vaccine immunogens that induce bNAbs? In practical terms, this approach requires using recombinant protein technologies to make spike‐mimetic immunogens, not the spikes themselves. To achieve that first goal requires an understanding of what the Env spike is, how it functions in the context of the HIV‐1 replication cycle, and what factors govern attempts to produce mimics that are capable of inducing NAbs, and ideally bNAbs.

## 2 The trimeric HIV‐1 Env spike as a basis for immunogen design

The HIV‐1 Env spike is a trimer of heterodimers. In other words, six individual protein subunits make up the assembled trimer. In an infected cell, the viral *env* gene is transcribed as a gp160 precursor polypeptide that is extensively glycosylated (see below) and then proteolytically cleaved by the serine proteinases of the Furin family into non‐covalently linked gp120 and gp41 subunits.^12^ Three of these gp120‐gp41 protomers assemble into the functional trimer, the Env spike. The presence of a hydrophobic membrane‐spanning domain as an integral component of each gp41 subunit anchors the trimer into the host cell membrane (which becomes the virus membrane after the particle is released). The gp120 subunits contain the CD4 receptor‐ and coreceptor‐binding sites and mediate the attachment of the spikes to target cells, while the gp41 subunits drive fusion of the virus and cell membranes.^13^, ^14^, ^15^ The spike is an unstable entity, reflecting its need to undergo profound conformational changes when functioning as a fusion machine. Thus, the sequential binding of the gp120 subunits to CD4 and a coreceptor drastically alters the conformation of these subunits, and releases their hold on a metastable conformation of gp41. The resulting structural rearrangements of gp41 domains liberate enough latent energy to drive the insertion of the fusion peptide into the host cell membrane, and thence virus‐cell fusion.^13^, ^14^, ^15^ The natural instability of the Env spike has substantial implications for its production as a vaccine antigen for bNAb induction strategies, as outlined further below.

## 3 The trimer hypothesis; why are trimers preferred immunogens?

As noted above, NAbs act to counter HIV‐1 infection by binding to the Env spike on the virus surface. By definition, then, NAb epitopes, including those for bNAbs, are present on the functional trimer. However, most antibodies that are raised against HIV‐1 Env proteins do not bind to the spike, or do so only under limited circumstances (e.g. to a subset of neutralization‐sensitive viruses). Such antibodies are generally referred to as non‐neutralizing antibodies (non‐NAbs). While they often have other antiviral activities, non‐NAbs are not the focus of this article (see review by Lewis & Devico in this volume^16^). Non‐NAbs are induced during HIV‐1 infection because multiple forms of Env co‐exist with the functional spike, arising from the inefficient processing and assembly of spikes and/or their later disintegration.^17^, ^18^, ^19^, ^20^, ^21^, ^22^ Furthermore, monomeric gp120 subunits that dissociate from spikes circulate as soluble proteins and the residual gp41 stubs are present on virus particles or infected cells.^17^, ^18^, ^22^ Such ‘viral debris’ is highly immunogenic, but the resulting antibodies tend to recognize only the debris and not the spikes themselves, where the relevant epitopes are either entirely absent or occluded by inter‐subunit interactions.^17^, ^18^, ^23^, ^24^, ^25^, ^26^ The extent to which the immunodominant epitopes present on viral debris are distractive from more desirable antibody responses is still, perhaps surprisingly, unresolved. However, some trimer‐based immunogen design strategies seek to minimize the induction of this category of non‐NAbs and focus the response on NAb epitopes.

Several sub‐categories of NAb epitope are present on the Env spike: Some spike epitopes are shared with simpler forms of Env such as gp120 monomers; some are present on gp120 monomers but their structure is substantially modified by trimerization; and some are completely specific to the native trimer.^27^, ^28^, ^29^, ^30^, ^31^, ^32^, ^33^, ^34^ The latter two classes are often referred to as trimer‐influenced and trimer‐dependent epitopes, respectively. Most bNAbs recognize epitopes that are, to various extents, influenced by the quaternary structure of the trimer, some exquisitely so. The latter, trimer‐dependent bNAb epitopes includes ones at the trimer apex and others at the interface between the gp120 and gp41 subunits.^27^, ^28^, ^29^, ^30^, ^31^, ^32^, ^33^, ^34^ Epitopes that are influenced by trimer formation but also present on gp120 monomers include ones associated with the CD4‐binding site (CD4bs). More specifically, the structure of the trimer constrains how such epitopes are presented. For example, the angle of approach that a bNAb can take to successfully encounter its CD4bs‐associated epitope on one protomer of the trimer can be limited by the presence of the other two protomers.^35^ The outcome is that although simpler forms of Env may present the same epitopes, they do not do so in a way that precisely mimics how they are oriented on the native spike.

In summary, the paramount argument that supports the development of recombinant trimer‐based immunogens is that trimers present multiple bNAb epitopes in ways that best mimic how these epitopes appear on the native Env spike.^23^, ^26^, ^36^ The presence of a bNAb epitope on an Env immunogen does not, of course, mean that bNAbs will be raised against it, but the absence of an epitope, or its presence in a conformationally inappropriate form, does reduce the chances of a good outcome.

## 4 Non‐native gp140 ‘pseudotrimers’

Early Env vaccine programs focused on the production of monomeric gp120 proteins, mostly based on sequences from laboratory‐adapted isolates (e.g. IIIB, MN, SF‐2). These gp120 monomers presented the NAb epitopes that were then known, principally ones against the V3 region and the CD4bs.^37^, ^38^ As immunogens, gp120 monomers proved unable to induce NAbs that were active against typically resistant (Tier‐2) viruses, and they failed to confer protection when tested in Phase III trials.^39^, ^40^, ^41^ Contributing to these outcomes may be the presentation of immunodominant non‐NAb epitopes, the inability to present NAb epitopes that depend on quaternary structure and/or involve gp41, and the generation of ‘off‐target’ non‐NAbs that approach the CD4bs on the trimer from an inappropriate angle. In addition, gp120 monomers are not always as simple to manufacture as is often thought, due to proteolytic damage to V3, unwanted dimerization via intermolecular disulfide bonds, and the formation of aberrant intramolecular disulfide bonds that can compromise the presentation of some epitopes.^42^, ^43^, ^44^, ^45^, ^46^, ^47^ We note that boosting of rabbits with gp120 monomers after priming with vaccinia viruses expressing gp160 induced a low but fairly consistent level of NAbs against heterologous Tier‐2 viruses.^48^

The limitations of gp120 monomers then drove multiple research groups to try to make trimeric mimics of the Env spike, that is, constructs that contained both the gp120 and gp41 subunits and were considered to be capable of adopting trimeric configurations. Because membrane‐associated proteins are generally harder to make and purify than secreted (i.e. soluble) ones, a generally adopted strategy involved truncating the gp41 subunit by adding a stop codon before the transmembrane domain. As a result, all the soluble gp140s contain gp120 subunits and the ectodomain of gp41 (gp41_ECTO_) but not the transmembrane and cytoplasmic regions.^49^ Eliminating the ‘unwanted’ regions of gp41 was soon found to adversely affect the stability of the already metastable trimer: the soluble gp140s rapidly dissociated into their constituent subunits, gp120 monomers and a trimerized form of gp41_ECTO_.^36^, ^49^ A very widely adopted, and seemingly successful, solution to the rapid loss of gp120 was to inactivate the REKR cleavage site between gp120 and gp41_ECTO_ by converting it into a sequence (e.g. SEKS) that was not a substrate for the Furin protease.^49^, ^50^ As a result, the gp120 and gp41_ECTO_ components remained covalently linked, allowing the purification of a gp140 protein of the right size (i.e. containing the desired number of each subunit, three). Yields of these trimers were often quite low, however, as the secreted Env proteins were highly heterogeneous.^51^, ^52^, ^53^ As well as the trimer fraction, gp140 monomers, dimers, and higher molecular weight aggregates were also present in substantial amounts, the latter now known to arise at least partially from the inappropriate formation of intermolecular disulfide bonds (see below).^54^, ^55^, ^56^, ^57^, ^58^ For reasons outlined below, from hereon we refer to the trimer fractions derived from the prototypic uncleaved gp140 constructs as ‘pseudotrimers’, while noting that many preparations may also have contained other Env species such as dimers and aggregates.

The introduction of an extraneous trimerization domain, most commonly the Foldon domain from the bacteriophage T4 fibritin protein or the isoleuzine zipper from the GCN4 transcriptional activator from *Saccharomyces cerevisiae*,^59^, ^60^ to the C‐terminus of gp41_ECTO_ improved the yield of the ‘trimer’ fraction and facilitated the production of immunogens for animal studies.^50^, ^61^, ^62^, ^63^, ^64^, ^65^, ^66^, ^67^, ^68^, ^69^, ^70^ The tendency for the postpurification formation of disulfide bond‐linked aggregates remained a factor, as did the long‐unappreciated problems associated with the scrambling of the native disulfide bond profile within the gp120 subunits that can compromise the presentation of relevant epitopes.^54^, ^55^, ^56^, ^58^, ^71^ In immunogenicity studies, any benefits of this generation of uncleaved gp140s, with or without Foldon stabilization, were modest, and restricted to small increases in the titers of NAbs to Tier‐1 viruses or of Env‐binding antibodies.^61^, ^64^, ^65^, ^66^, ^67^, ^68^ The Foldon and GCN4 trimerization motifs were also found to be highly immunogenic, although the implications are unknown.^71^ Tier‐2 NAbs, even to autologous viruses, were rarely induced, exceptions being a study in which macaques were co‐immunized with a DNA plasmid expressing gp160 and a gp140 pseudotrimer,^72^ and a study in which macaques were immunized seven times with a gp140 pseudotrimer.^73^ When pseudotrimers had been tested in humans, the NAb responses were weak and limited to Tier‐1 viruses.^74^, ^75^, ^76^, ^77^, ^78^

The limitations of these uncleaved gp140 constructs only became understood more than a decade after they were first developed, when new analytical techniques and reagents became available over the period of a few years.^26^, ^54^, ^55^, ^56^, ^58^, ^71^, ^79^, ^80^, ^81^ A new generation of bNAbs to trimer‐dependent or ‐influenced epitopes served as important guides for the antigenic properties of trimer‐mimicking Env proteins. Thus, Env proteins that were unable to bind bNAbs such as PGT145 or PGT151 efficiently were not properly presenting key epitopes that have become hallmarks of the native trimer.^58^, ^71^, ^81^ At around the same time, negative‐stain electron microscopy (NS‐EM) methodologies were developed that allowed gp140 proteins to be visualized in enough detail (approximately 20 Å resolution) to reveal how the individual subunits were oriented.^57^, ^58^, ^71^, ^80^, ^82^, ^83^, ^84^ The power of these reagents and techniques was amplified when native‐like SOSIP trimers were developed that could serve as comparators (see below). Thus, although NS‐EM imaging of the SOSIP trimers showed they adopted homogenous, tri‐lobed, propeller‐shaped configurations, the uncleaved gp140s of the same or different genotypes had a very different appearance, whether they contained Foldon domains or not. An eclectic gallimaufry of shapes was visible, often with three spatially separated gp120 subunits linked to a smaller, central core comprising the trimerized gp41_ECTO_ components.^57^, ^58^, ^71^, ^80^, ^82^, ^83^, ^84^ Why bNAbs to quaternary epitopes were unable to bind these pseudotrimers was now obvious from the imagery. Multiple additional analytical techniques, including hydrogen–deuterium exchange, glycan composition, disulfide bond profiling, and thermal dissociation measurements have confirmed the profound differences between the pseudotrimers and truly native‐like trimers.^56^, ^57^, ^58^, ^71^, ^79^, ^80^, ^82^, ^83^, ^84^ Nonetheless, some pseudotrimers are still being tested in clinical trials, presumably based around their abilities to induce possibly protective non‐NAbs.^57^, ^69^, ^85^ More recent designs of uncleaved gp140s that do form native‐like trimers are discussed further below.

## 5 The development of native‐like trimers

**Figure 1:**
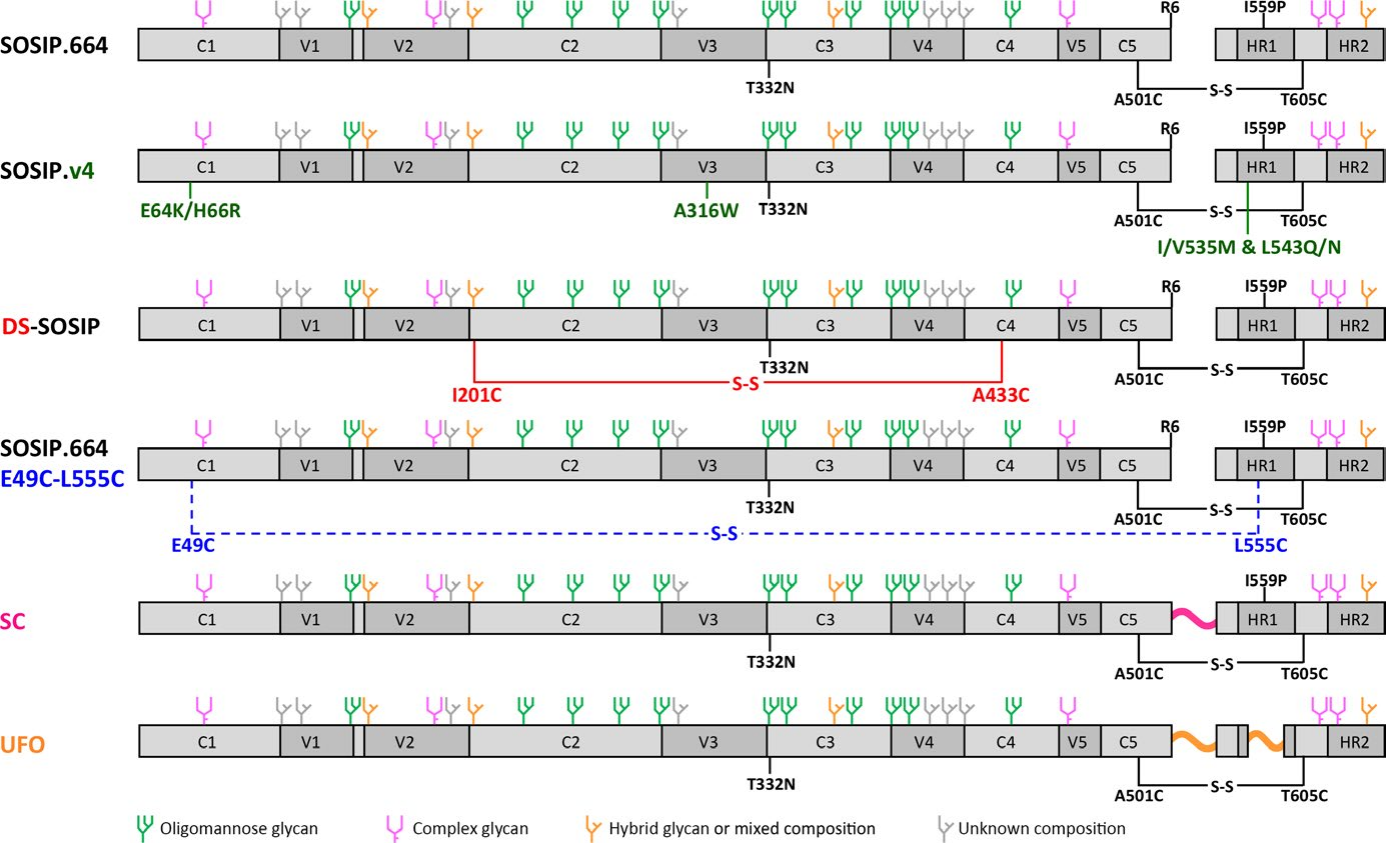
Schematic presentations of various native‐like trimer constructs. Linear schematic of the SOSIP.664, SOSIP.v4, DS‐SOSIP, SOSIP.664 E49C‐L555C, SC, and UFO gp140 constructs that are mentioned in the text. Each schematic is based on the BG505 genotype, with the conserved (C) and variable (V) domains of gp120 and the two heptad repeats (HR) of gp41_ECTO_ depicted in gray. The locations of N‐linked glycans are indicated above the construct bars. The N332 glycan that was knocked into the BG505 gene used to make every construct is highlighted in black (T332N). The assignment of the various glycans to the oligomannose (green), complex (purple), or mixed or hybrid (orange) type is based on data derived using BG505 SOSIP.664 trimers.^124^ The glycan site‐specific composition of the other trimers has not been determined and may differ to various extents from what is depicted. The SOS bond (501C‐605C) linking gp120 to gp41_ECTO_ and the I559P substitution in gp41_ECTO_ (not included in the UFO construct) are shown in black, as is the RRRRRR (R6) change to the Furin cleavage site (not present in the SC and UFO designs). The additional disulfide bonds engineered into the DS‐SOSIP and SOSIP.664 E49C‐L555C constructs are represented by solid red and dotted blue lines, respectively. The point substitutions, compared to SOSIP.664, that are present in the SOSIP.v4 constructs are shown in green (SOSIP.v4.1, E64K; SOSIP.v4.2, H66R).^123^ The flexible linker between gp120 and gp41_ECTO_ in the SC construct is indicated by an undulating purple line, and the same linker as well as an additional one in HR1 (HR redesign) that is present in the UFO construct are both in orange.^128^, ^132^ The NFL construct is essentially identical to the SC design except that it does not include the SOS bond and the flexible linker length varies slightly.^132^ All constructs terminate at residue 664^102^

An alternative to eliminating the cleavage site between gp120 and gp41_ECTO_ when making soluble trimers was to retain the site and then address the resulting instability consequences. The rationale for keeping the cleavage site was that separating the gp140 protein into its constitutive subunits seemed likely to have consequences at the structural level. To ensure that as many gp140 proteins as possible were cleaved efficiently, the producer cell's natural of supply of Furin‐like proteases was supplemented by co‐transfecting the *furin* gene simultaneously with one encoding the gp140 construct.^36^ Moreover, the natural REKR cleavage site was later optimized by mutagenesis to create the more scissile RRRRRR (R6) motif^86^ (Fig. 1).

**Figure 2:**
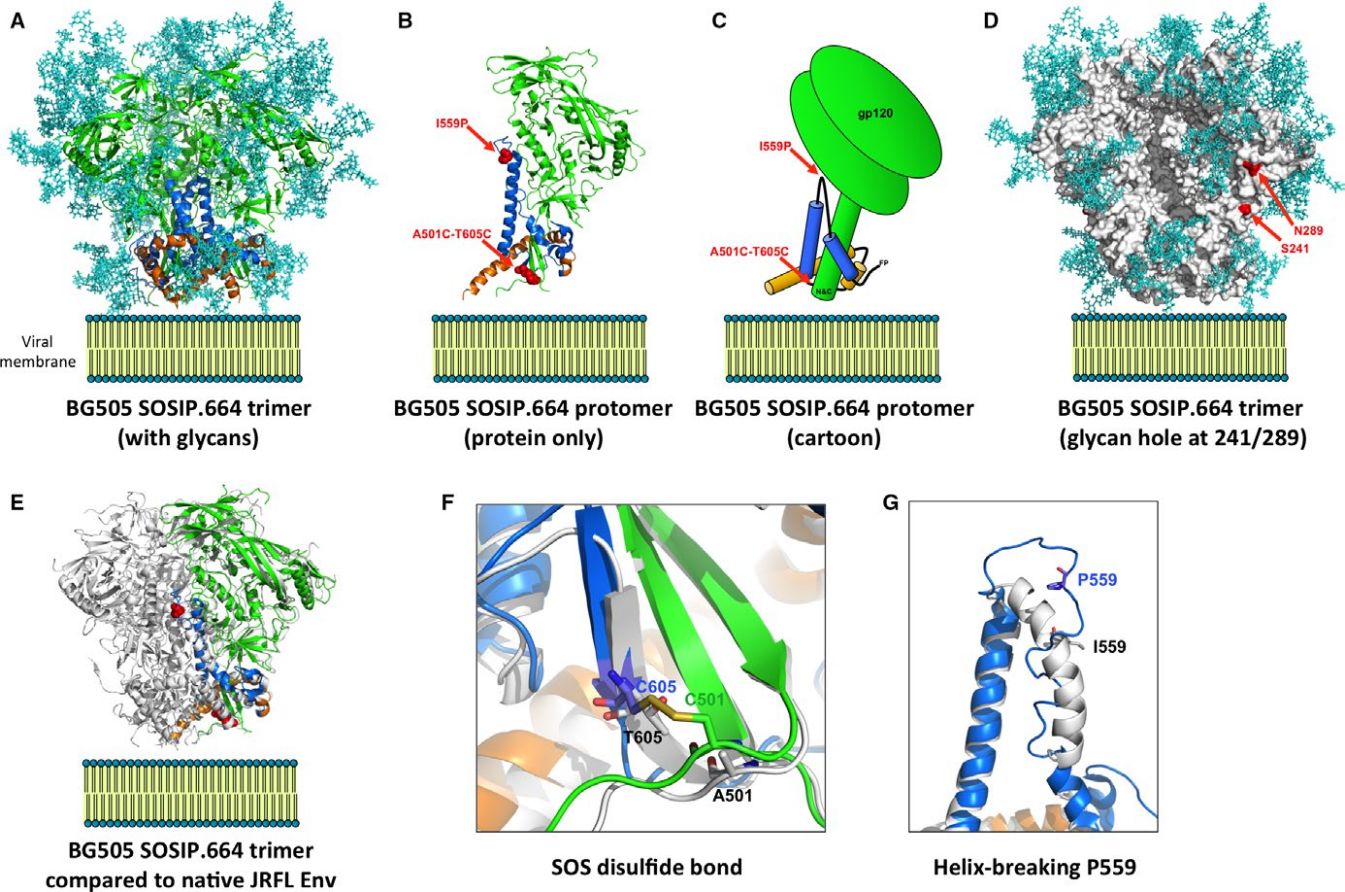
Structure and glycosylation of the BG505 SOSIP.664 trimer and comparison with the membrane‐associated native JR‐FL trimer. (A) Structure of the BG505 SOSIP.664 trimer (4TVP)^107^ with the gp120 subunits in green and gp41_ECTO_ in blue, except for HR2 (orange). The position of the viral membrane is shown for orientation. The glycans were attached to the protein using GlyProt (http://www.glycosciences.de/glyprot/) according to the composition determinations described in Behrens et al.^124^ For glycans where the composition could not be determined in that study, we used information from other reports.^126^, ^212^, ^213^, ^214^ Overall, Man_9_GlcNAc_2_ glycans are shown at positions N156, N160, N234, N262, N276, N295, N332, N339, N363, N386, N392, and N448; Man_5_GlcNAc_2_ glycans at positions N197, N301, N355, and N411; and fucosylated biantennary complex glycans at position N88, N133, N137, N462, N611, N618, and N637. We note however, that the composition of many glycans is heterogeneous.^124^ We did not add a glycan to residue N625 because that position may be only partially occupied.^126^ We were unable to add glycans to positions 190, 190c, 398, and 406 because these asparagine residues were not resolved in the structure and hence are not included in the model. (B) Structure of one BG505 SOSIP.664 protomer (5CEZ)^114^ with the same color code as in panel‐A. Residues P559, C501, and C605 are shown as red spheres. (C) A cartoon rendering of panel‐B. (D) The same structure as in panel‐A is shown, but with the protein component in white as a space‐filling representation and with residues 241 and 289 (i.e. the location of the ‘glycan hole’) shown in red and indicated by arrows. (E) The protein structure of one BG505 SOSIP.664 protomer (as in panel‐B) is overlaid with the structure of the native, membrane‐associated JR‐FL trimer in white.^89^ (F) Detail of the overlay from panel‐E showing positions A501 and T605 in the JR‐FL trimer together with the cysteine substitutions (C501 and C605) and the linking disulfide bond that are present in the BG505 SOSIP.664 trimer. (G) Detail of the overlay from panel‐E showing part of gp41 and the effect the I559P substitution has on the local structure

The most obvious manifestation of the fragility of cleaved gp140 proteins was the immediate dissociation of gp120 from gp41_ECTO_.^36^ To overcome that problem, the introduction of appropriately positioned cysteine residues (501C‐605C; referred to as SOS) created an engineered disulfide bond between the two subunits^36^ (Fig. 1). The location of the gp41 cysteine residue was based on where one is positioned in the ectodomains of the transmembrane proteins of many mammalian retroviruses. Thus, all these proteins contain a similarly positioned, short disulfide‐bonded loop (CxxxxxxCx), but in type‐C and type‐D retroviruses such as MLV and HTLV‐1, an additional cysteine located immediately adjacent to the C‐terminal end of the above motif (i.e. CxxxxxxC**C**) had been proposed to play a role in inter‐subunit linkages.^87^ Of note was that the ‘third cysteine’ was absent from all lentiviral transmembrane proteins. It seemed an obvious choice for engineering a covalent linkage between gp120 and gp41_ECTO_. The location of the counterpart cysteine residue in gp120 was less obvious, but the choice was guided by a mutagenesis study implicating short stretches of the gp120 C1 and C5 regions as the site(s) that interacted with gp41.^88^ A trial and error approach enabled the identification of residue‐501 as the most suitable site for the engineered cysteine substitution. Over 15 years later, the high‐resolution structure of the native trimer confirmed the appropriateness of this choice^89^ (Fig. 2F).

The resulting SOS gp140s were still highly unstable, dissociating into gp120‐gp41_ECTO_ monomers because of the propensity of the gp41 components to undergo substantive conformational transitions toward the postfusion configuration.^90^, ^91^ The introduction of a helix‐breaking point substitution, I559P, in heptad repeat 1 (HR1) that was intended to prevent such structural changes had the desired effect (Fig. 2G), allowing the gp41_ECTO_ components to remain in their prefusion, ground state^90^, ^92^ (Fig. 1). The resulting construct, then designated SOSIP gp140, yielded trimers efficiently, as judged by the then available analytical techniques.^90^

The early SOSIP trimers were based on the JR‐FL clade B sequence, which was chosen because the JR‐FL virus was a widely studied, CCR5‐using, neutralization‐resistant virus. In retrospect, it is now clear that the JR‐FL sequence forms native‐like soluble trimers quite poorly,^93^ but that knowledge required the development of better analytical techniques (e.g. NS‐EM, see above). An empirical approach based on testing a range of *env* genes revealed that the KNH1144 clade A sequence formed SOSIP trimers more efficiently than JR‐FL, a development that allowed useful, intermediate resolution structural information to be obtained.^94^, ^95^, ^96^, ^97^, ^98^, ^99^ Some changes to the gp41 sequence were found to improve trimerization.^100^, ^101^ The advent of NS‐EM methodology identified a problem with the KNH1144 SOSIP trimers and also suggested a solution; the hydrophobic MPER at the C‐terminus of gp41_ECTO_ had a tendency to cause trimers to aggregate, and it also acquired lipids from the culture medium and/or producer cell. The resulting limitations to the structural utility and possibly to the immunogenicity of the trimers were overcome by truncating the MPER to create a more soluble version. The new design was named SOSIP.664, as the C‐terminal residue was now at position 664 instead of its previous location, position 681.^102^, ^103^ The loss of the MPER bNAb epitopes seemed a price worth paying for a higher quality product.

The next design evolution came from another *env* gene‐screening program, which was set up to identify sequences that yielded SOSIP.664 trimers at an improved yield. The outcome was the BG505 SOSIP.664 construct, which became the prototypic native‐like soluble trimer^26^ (Fig. 1). The BG505 clade‐A sequence was derived from a 6‐week‐old Kenyan infant that had become HIV‐1 infected at birth^104^, ^105^; the BG505 virus is therefore a transmitted/founder, and it has the typical R5 and Tier‐2 phenotypes. Of note is that the infant developed a bNAb response over the next 27 months, as discussed further below.^105^

## 6 Properties of BG505 SOSIP.664 trimers

The BG505 SOSIP.664 trimer could be purified in good yields from 293T/F cell transient transfection supernatants by the use of a 2G12 bNAb affinity column followed by size exclusion chromatography (SEC), a procedure that we and others now use routinely for making multiple different trimers.^26^, ^83^, ^84^, ^106^, ^107^, ^108^, ^109^, ^110^ When viewed by NS‐EM, the 2G12/SEC‐purified BG505 trimers were highly homogeneous, with >95% having the regular, tri‐lobed propeller shape that is now seen as a defining characteristic of native‐like soluble trimers.^26^ The trimers were also highly stable, with melting temperatures of approximately 67°C in thermal stability assays. Antigenically, the BG505 SOSIP.664 trimers had the highly desirable property of binding all known bNAbs (except, obviously, ones against the MPER that had been truncated). Conversely, the trimers bound non‐NAbs (for Tier‐2 viruses) very poorly or not at all, including those to CD4‐induced and non‐neutralizing CD4bs epitopes. In some assays (e.g. ELISAs using epitope‐tagged trimers), the V3 region of the trimers was antigenic for non‐NAbs, but much less so in other test systems (e.g. SPR, ITC, Octet, and NS‐EM).^26^, ^81^, ^111^ Overall, the antigenic properties of the BG505 SOSIP.664 trimers closely reflected those of the native BG505 Env spike, as judged by a strong correlation between the outcomes of trimer‐binding and virus‐neutralizing assays using a panel of NAbs and non‐NAbs.^26^ The development of stable 293T and CHO cell lines expressing trimers of comparable quality but in higher yields increased the supply and facilitated multiple in vitro and then immunogenicity studies.^106^

The BG505 SOSIP.664 trimers soon proved to be valuable for structural studies, including ones aimed at characterizing known and new bNAbs. The first authentic high‐resolution structures of a HIV‐1 Env trimer were obtained by two methods, the traditional x‐ray crystallography approach and cryo‐EM.^35^, ^112^ In both cases, the trimers were complexed with bNAbs, PGT122 to a V3‐glycan epitope for the x‐ray structure, PGV04 to the CD4bs for cryo‐EM. The two structures, at resolutions of 4.7 and 5.8 Å, respectively, were highly concordant. Another x‐ray structure, which revealed the gp41 subunits at slightly higher resolution, was soon obtained independently using the same BG505 SOSIP.664 trimers as a ternary complex with bNAbs PGT121 and 35O22^107^ (Fig. 2A). Several additional SOSIP trimer structures of the same and different genotypes have since been obtained, a topic that will be reviewed in more detail in another article^110^, ^113^, ^114^, ^115^, ^116^ (reviewed in Ref. 117 in this volume; Fig. 2). These cumulative insights have increased our knowledge of the inherent, and necessary, metastability of the trimer, and how its gp120 and gp41 components function in concert after receptor engagement to drive HIV‐1 entry into cells^14^, ^35^, ^107^, ^108^, ^110^ (Fig. 2). Of note is that the high‐resolution cryo‐EM structure of full‐length, membrane‐derived BG505 trimers with no SOSIP substitutions is essentially identical to the soluble BG505 SOSIP.664 trimer structure^89^ (Fig. 2E,F); the only exception is the region around residue 559, where the proline substitution in the SOSIP.664 trimer breaks a helix, in the way it was designed to do^89^, ^90^, ^92^ (Fig. 2G).

Multiple bNAb epitopes have been identified and/or characterized using BG505 SOSIP.664 trimers. The trimers greatly facilitated the definition of the PG9 epitope, which binds the trimer apex with an unusual 1:1 stoichiometry that was not apparent from earlier studies with gp120 monomers.^118^ A new cluster of quaternary structure‐specific epitopes was mapped to the gp120‐gp41 interface, firstly PGT151 and then 35O22, VRC34, 3BC315, and 8ANC1195.^27^, ^28^, ^30^, ^31^, ^32^, ^33^, ^34^ The use of labeled BG505 SOSIP.664 trimers for sorting Env‐specific B cells identified several new bNAbs, including the extremely high‐affinity PDGM1400 bNAb against the trimer apex,^31^ potent variants from the VRC26‐lineage^119^ and ACS202 against the gp120‐gp41 interface.^34^ One striking cumulative finding is that almost the entire surface of the trimer contributes to one or more bNAb epitopes. Thus, contrary to earlier perceptions, there are not just a few areas of vulnerability on the trimer, there are many; the human immune system has found a range of different solutions to the problems of generating bNAbs against the epitopes presented on the trimer.^120^

The BG505 SOSIP.664 trimer also enabled a better understanding of how Env proteins were folded and glycosylated, particularly when comparative studies were performed using gp120 monomers and gp140 pseudotrimers. For several years it had been apparent, although generally underappreciated, that these proteins, of various genotypes, were affected by the inappropriate formation of non‐canonical disulfide bonds.^43^, ^45^, ^47^, ^54^, ^55^ In other words, in a substantial proportion of the total population of gp120 monomers or subunits some of the intermolecular disulfide bonds had become scrambled because the ‘wrong’ cysteines had paired up. The resulting gp120 molecules, although of the correct size, were misfolded and antigenically perturbed, particularly within the V1V2 region where multiple cysteines are located.^47^, ^54^, ^55^ In addition, the aberrant formation of intermolecular disulfide bonds yielded dimerized gp120 proteins.^43^, ^45^, ^47^ For the gp140 pseudotrimers, the problem was similar but generally worse: aberrant disulfide bonds were present in a variable but often substantial population of the gp120 subunits, disulfide cross‐linking formed gp140 aggregates, and it seems likely that the three gp120 subunits could also be internally cross‐linked by the same mechanism.^54^, ^55^, ^56^, ^58^, ^71^ In retrospect, disulfide cross‐linked Env proteins could be easily detected on non‐reducing versus reducing SDS‐PAGE gels, an analysis that was often not performed or not shown in early reports.

In contrast, comparative analyses showed that SOSIP.664 trimers were almost entirely unaffected by the disulfide‐scrambling problem.^26^, ^56^, ^58^, ^71^ The likely explanation is that the formation of inappropriate disulfide bonds leads to misfolded subunits that simply cannot pack together properly when a trimer assembles within the producer cell. Then, once a stable trimer has formed, its disulfide bonds are sterically shielded from the actions of protein disulfide isomerases that can act on the more accessible bonds present in gp120 monomers or gp140 pseudotrimers.

The steric constraints imposed by trimer formation also account for the major differences in glycan composition seen when SOSIP.664 trimers were compared with gp120s and gp140 pseudotrimers. Thus, native‐like trimers have a much higher content of oligomannose glycans^83^, ^121^, ^122^, ^123^, ^124^ (Fig. 2A), which is relevant because these structures mimic those of native Env and contribute to the epitopes for multiple classes of bNAbs. In contrast, the glycans on gp120 monomers and gp140 pseudotrimers are more processed, creating structures that are less or not compatible with bNAb epitopes.^55^, ^58^, ^83^ The underlying reason is that trimer formation impedes the accessibility of mannosidase enzymes to their substrates at many positions, which leaves the trimer in a mannose‐rich form. However, the same enzymes can gain access to the more exposed glycan moieties on the gp120s and gp140s, trimming away the mannoses and creating substrates for other glycan‐modifying enzymes that then produce complex, non‐native structures at multiple sites. Because enzyme access to glycans varies across the surface of native trimers, some glycans end up in highly processed forms while others remain as oligomannose structures. A landmark analysis of the glycans present at the different sites on the BG505 SOSIP.664 trimer showed that a large patch of closely grouped oligomannose glycans encircles the gp120 outer domains, with more highly processed structures present at the apex and, particularly, the stem of the trimer where the steric constraints on enzyme access are diminished.^124^

## 7 Improving the properties of BG505 and other SOSIP.664 trimers

In contrast to BG505, NS‐EM imaging showed that many Env sequences did not yield fully native‐like SOSIP.664 trimers. Often, the 2G12/SEC‐purified trimers were a mixture of native‐like and non‐native forms, the latter morphologically resembling gp140 pseudotrimers. Nonetheless, screening assays that included NS‐EM endpoints identified several additional SOSIP.664 trimers with properties similar to BG505 (i.e. approximately 100% native‐like), including the clade B trimer B41 and the clade C trimers DU422 and ZM197M.^108^, ^109^ The development of positive‐selection columns based on trimer‐specific bNAbs increased the range of Env sequences from which native‐like trimers could be generated (see below). A greater awareness of the sequence influences on native‐like trimer formation has also helped to further increase the generality of the method, via targeted mutagenesis.

Several interlinked aspects of the SOSIP technology required further improvement. First, since native‐like trimers based on just one isolate (e.g. BG505) were probably not sufficient to meet the goal of improving NAb breadth, ensuring that the method was generalizable became a high priority. This was not trivial because Env sequences can differ greatly in their propensity to yield native‐like SOSIP trimers efficiently. Second, considering the complex nature of bNAb epitopes, any local or global instability and flexibility may compromise the ability of trimers to engage naive B cells that have the capability to evolve into bNAb‐producing cells. Third, the exposure of immunodominant non‐NAb epitopes such as V3 might interfere with the induction of more desirable specificities. In this section, we describe several often complementary strategies to improve SOSIP trimers: (i) by design changes (i.e. by mutation); (ii) by the adoption of alternative purification methods; (iii) by chemical modification. Many of these changes can and have been applied to alternative designs of native‐like trimers.

One perceived weakness of the original SOSIP.664 design was that V3 non‐NAb epitopes could become exposed under certain conditions in vitro as the fully closed trimer transits to a more open form (see above). V3 exposure must also take place under in vivo conditions, because V3 is highly immunogenic when SOSIP.664 trimers are used to immunize rabbits.^84^, ^123^ This type of conformational transition is probably related to the natural process of trimer ‘breathing’.^125^, ^126^, ^127^ It might have no adverse consequence for the induction of NAbs, but an alternative perspective is that the exposure of V3 and associated non‐NAb epitopes may impede the presentation of key epitopes associated with the fully closed configuration. Furthermore, the immunogenicity of non‐NAb epitopes, including but not limited to V3, might compromise the induction of NAbs to more relevant epitopes by any of various immunological distraction mechanisms^111^, ^123^ (and references therein). As it was and remains impossible to adjudicate between these various scenarios a priori, various approaches to further stabilizing the SOSIP.664 trimer were initiated. As well as perhaps improving immunogenicity, an additional goal of the stabilization projects was to increase the range of genotypes that would form fully native‐like trimers; the assumption was that inherently unstable trimers were more likely to decay into non‐native conformations or even disintegrate into their constituent gp140 protomers.

Before the identification of BG505‐based trimers, a comparison of the KNH1144 sequence with JR‐FL, which formed trimers less efficiently, identified various residues in the HR1 of gp41_ECTO_ as beneficial to trimerization.^100^, ^101^ A sequence comparison with BG505 confirmed the importance of some of the residues, most notably M535 (compared to I535) and Q543 or N543 (compared to L543).^123^ These residues were shown to improve the trimerization propensity and quality of SOSIP proteins based on isolates B41, AMC008, AMC011 (all clade B), and ZM197M (clade C), and are now routinely incorporated into next‐generation designs such as the SOSIP.v4 constructs^34^, ^123^ (Fig. 1).

Another reported stabilization modification to the BG505 SOSIP.664 trimer involved the structure‐guided introduction of an additional disulfide bond within each gp120 subunit to create the DS‐SOSIP variant.^110^ Specifically, the new bond, created by cysteine point substitutions, linked residue 201 in C2 with residue 433 in C4 (Fig. 1). The resulting trimer was more stable, as shown by an increase in its melting temperature from 67.0°C to 73.1°C, and more rigid in an HD‐X assay. The binding of CD4 and the induction of CD4‐induced conformational changes were both reduced, as was the exposure of V3 non‐NAb epitopes.^110^ Based on a new 3.0‐Å structure of the BG505 SOSIP.664 trimer that revealed additional details of the gp41 HR1 domain, an inter‐protomer disulfide bond was introduced between residue 49 in the C1 domain of gp120 and residue 555 in HR1.^114^ That bond provided additional stability by cross‐linking individual protomers, and raised the trimer melting temperature to 75.2°C. It has not yet been reported whether these two ways to increase stability and improve antigenicity translate into superior immunogenicity.

An alternative approach to the same design issues involved the introduction of two substitutions in C1 (E64K or H66R) and V3 (A316W). The E64K and H66R substitutions were guided by studies on infectious virus mutants that revealed how the two changes prevented the spontaneous transition of the native trimer to the CD4‐bound conformation. They were applied to the SOSIP.664 trimer with the goal of reducing its propensity to undergo conformational transitions to the more open configuration associated with CD4 binding, and hence the exposure of CD4i non‐NAb epitopes.^123^ The structure‐guided introduction of the A316W substitution was designed to increase inter‐domain hydrophobic interactions within gp120 and thereby impede the movement of V3 and its associated non‐NAb epitopes into a more exposed conformation.^123^ The new trimer variants were designated SOSIP.v4.1 (containing E64K and A316W) or SOSIP.v4.2 (containing H66R and A316W) (Fig. 1); they had the desired antigenicity properties, were more thermally stable (from 67.0°C to 69.5°C and 69.3°C, respectively) and were more rigid when assessed by HD‐X. The stabilization method was general applicable because the same substitutions had similar effects on trimers from multiple genotypes, including ones from clade A (BG505), clade B (B41, AMC008, AMC011), and clade C (ZM197M).^34^, ^123^ Immunogenicity studies of SOSIP.v4 trimers in rabbits are summarized below.

Additional alterations to part of HR1 have some beneficial effects on the production and antigenic properties of the resulting BG505‐based trimers.^128^ The high‐resolution trimer structures suggest that a bend spanning residues G547 and T569 within HR1 is poorly ordered. This bend connects the long central helix to the fusion peptide, and includes the helix‐breaking I559P substitution that was introduced to stabilize the original SOSIP trimer design^90^, ^92^, ^128^ (Figs 1 and 2G). Computational biology techniques identified ways to redesign this stretch of HR1, by replacing the original sequence with 8‐ or 10‐residue loops. In the context of the BG505 sequence, the resulting constructs, designated ‘HR1 redesigns 1 and 2’, yielded trimers with broadly similar properties to SOSIP.664, with a modest increase in trimer formation (i.e. fewer dimers and aggregates prior to the trimer purification step) and a small reduction in the exposure of various non‐NAb epitopes on the purified trimers. The methodology was then applied to other trimer genotypes (JR‐FL, DU172.17, CH115.12, and CN54) that were known to produce trimers less efficiently than BG505. When a ‘generic’ HR1 linker sequence was introduced into these four sequences, minor increases (<1.5‐fold) in the yield of the trimer fraction compared to the SOSIP.664 design were sometimes seen.^128^ The HR1 redesign method was also applied to the uncleaved, linker‐stabilized uncleaved prefusion optimized (UFO) trimer construct (see below; Fig. 1).

Alternative or complementary approaches to improve the quality of native‐like Env trimers involve new purification strategies. As noted above, our standard procedure for purifying SOSIP trimers was initially via 2G12 affinity columns followed by SEC. However, a variant technique that we have found to be very valuable is based on the observation that a subset of bNAbs, such as PGT145 and PGT151, recognize only native‐like trimers and not non‐native Env species.^26^, ^27^, ^28^, ^120^, ^129^ Affinity chromatography using PGT145 and PGT151 thus selects only pure, native‐like trimers from the input mixture of multiple Env forms. Positive selection on a PGT145 column has been used to obtain homogeneous native‐like SOSIP trimers based on isolates B41, AMC008, AMC011, and 92UG037.^34^, ^58^, ^109^, ^123^ Similarly, a PGT151 column positively selected native‐like CZA97 SOSIP trimers.^58^ For routine purposes, fully native‐like trimers can often be purified in a ‘one‐step process’ via PGT145 or PGT151 columns, although we sometimes find it advisable to also use SEC to remove a small amount of aggregated material. The successful purification of BG505‐based trimers on VRC01 bNAb affinity columns has also been reported, together with adaptations of that method.^107^, ^116^

Conversely, negative selection columns based on non‐NAbs (e.g. F105 or GE136 against the CD4bs) have been used to remove non‐native Env forms from JR‐FL and 16055 SOSIP.664 trimer preparations^93^ and likewise for several single‐chain, (SC) native‐like trimers (see below).^130^, ^131^ Of course positive and negative selection methods both incur a yield penalty. Thus, when used to find the proverbial needle in a haystack, they may yield trimers of high quality but in too little quantity to be of practical use. It remains critical to design constructs that have a high propensity to form native‐like trimers; only then can an appropriately chosen affinity column be used to clean up the initial preparation. Finally, SOSIP trimers that incorporate a C‐terminal His‐tag can be purified using Ni‐NTA columns, and similarly for other added purification tags.^82^, ^83^ One limitation of this conformationally non‐selective method is that it only works well with constructs such as BG505 that form native‐like trimers efficiently. In other cases, the presence of the purification tag on multiple forms of Env, particularly non‐native trimers, can compromise its value. Lectin affinity columns are also used to purify trimers via glycan binding.^93^, ^128^, ^130^, ^131^, ^132^, ^133^ We have occasionally used this method, but do not favor it from the product‐quality perspective.^108^ It is also possible that lectin columns may select for trimers with a different glycan composition compared to those purified on bNAb columns, which would need to be assessed experimentally.

Trimers can be chemically modified to decrease their conformational flexibility and improve their stability and antigenicity properties. The concept originates in an observation made over 20 years ago that chemical cross‐linking of virion‐associated Env proteins by formalin was compatible with the preservation of NAb epitopes.^134^ Since then, various groups have reported on the cross‐linking of cell surface‐associated Env, gp120 monomers and gp140 pseudotrimers,^135^, ^136^, ^137^, ^138^ before the originators of the cross‐linking concept then applied their method to native‐like trimers.^139^ Thus, treating BG505 or B41 SOSIP.664 trimers with glutaraldehyde or a hetero‐bifunctional cross‐linker further stabilized them with only minimal adverse effects on antigenicity. More specifically, the binding of most bNAbs was preserved after cross‐linking, while non‐NAb reactivity was reduced. All the Env conformers present within a mixed population were, of course, cross‐linked. However, the desired native‐like trimers could be purified by positive selection on PGT145, PGT151, or 3BC315 affinity columns or via negative selection with a non‐NAb against the V3 region.^139^ Of note is that the chemical cross‐linking of BG505 and B41 SOSIP.664 trimers improves their abilities to induce autologous Tier‐2 NAbs in rabbits (Q. Sattentau, personal communication; immunogenicity studies, in general, are discussed below). The glutaraldehyde cross‐linking method has also now been applied to the native‐flexible‐linker (NFL) design of JR‐FL and BG505 native‐like trimers (see below), with broadly similar outcomes.^131^

## 8 Alternative designs of native‐like trimers

The observations that gp140 cleavage was critically important for the formation of native‐like trimers were best explained by the argument that, without cleavage, the gp120‐gp41 interface did not form completely.^71^ Thus, critical gp120 residues, probably in the C5 region, that interact with their counterparts in gp41 to form the inter‐subunit‐binding site were unable to do so when gp120 remained covalently linked to gp41 in the uncleaved gp140s. The resulting loss of stability at the gp120‐gp41 interface means that gp120 either never associates properly with gp41 or rapidly dissociates from it; in either case, the gp120 subunits remain covalently tethered to gp41 via the uncleaved peptide but not in a way that mimics the structure of the native trimer. Accordingly, the quaternary structure‐dependent epitopes for bNAbs like PGT145 and PGT151 are absent from these constructs.^27^, ^28^, ^58^, ^71^, ^81^ Moreover, as discussed above, there are other serious consequences for antigenicity, disulfide bond formation, and glycan composition.

The question then arose as to whether the gp120‐gp41 interface could form properly without the necessity for Furin cleavage. The driving force for addressing this question was the perception that it was disadvantageous to have to co‐transfect the *furin* gene when making SOSIP trimers. Although Furin co‐transfection is inexpensive and technically simple, particularly via a single plasmid that expresses both *env* and *furin*,^106^ it was argued that there might be a reduction in trimer yield. An additional rationale was that eliminating the need for Furin cleavage could simplify Env immunization strategies based around DNA, mRNA, viral vectors, or self‐assembling nanoparticles.^128^, ^130^, ^132^, ^140^, ^141^ Four groups each followed the same strategy of adding a flexible Gly‐Ser linker sequence of between 8 and 20 residues between the gp120 and gp41_ECTO_ components of the gp140 construct, to replace the Furin cleavage motif.^69^, ^128^, ^130^, ^132^ The rationale was to eliminate the constraints on the gp120 C5 region and allow key residues to find their counterparts on gp41. The first report of this flexible linker approach showed that the resulting CZA97 and UG037 constructs had, in effect, identical properties to the standard uncleaved gp140‐Foldon pseudotrimers of the same genotypes.^69^ As the latter were clearly non‐native, this finding was apparently not supportive of the utility of the flexible linker approach.^58^, ^69^ Three later papers, however, showed that the flexible linker method could work, but only when it was used in conjunction with the I559P change and, for some genotypes, the SOS bond that are central features of the SOSIP design.^58^, ^128^, ^130^, ^132^

The SC gp140 constructs were the first next‐generation uncleaved gp140s that adopted a consistently native‐like conformation, as judged by antigenicity and NS‐EM criteria after purification on VRC01 columns followed by SEC.^130^ The construct was based on the BG505 sequence, included both the I559P and SOS changes present in SOSIP trimers and was truncated at residue‐664 (Fig. 1). Thus, in effect, the BG505 SC‐and SOSIP.664 constructs were identical except at the cleavage site between gp120 and gp41_ECTO_ where the flexible GlySer linker replaced the optimized Furin motif, RRRRRR. An investigation of the length for the flexible linker (1–20 residues) showed that the optimal sequence was GGSGGGGSGGGGSGG (i.e. 15 residues). From an antigenicity perspective, the SC‐gp140 variants were highly similar to SOSIP.664, the most notable difference being the loss of the gp120‐gp41_ECTO_ interface epitope for PGT151. In all other regards, the two constructs were essentially indistinguishable biochemically or by NS‐EM.^130^

The NFL gp140 design was very similar to that of its earlier SC‐gp140 counterpart, except that the SOS bond was omitted 132 (Fig. 1). This design was applied to the BG505 and JR‐FL isolates. For JR‐FL a negative‐selection purification using non‐NAb F105 was required to eliminate a dominant population of non‐native proteins before the native‐like trimer fraction could be studied. Flexible linker lengths of 5‐, 10‐, and 15‐residues were analyzed and the chosen length of 10‐residue (i.e. GGGGSGGGGS) residues was reasonably consistent with the outcome of the optimization study were in the earlier report on the SC‐gp140 design summarized above. Overall, the NFL‐ and SC‐gp140s appear to have similar properties in vitro, which reflects the similarities in their designs.^130^, ^132^ The NFL gp140s further benefited from a comparative sequence analysis involving BG505 and JR‐FL, focusing on the gp120‐gp41_ECTO_ interface and guided by high‐resolution structural information.^133^ This analysis flagged a number of residues that seemed likely to improve trimer information. Transfer of 8‐ or 15‐residues (termed the TD8 and TD15 constructs, respectively) from BG505 to the JR‐FL and 16055 genotypes of NFL trimer then supported the predictions of the in silico analysis.^133^

As noted above, the initial test of the flexible linker concept, using the CZA97 and UG037 genotypes, did not yield native‐like trimers.^69^ A follow‐up study both confirmed and solved the original design flaw. Thus, when the flexible linker was accompanied by both the I559P and SOS changes, native‐like CZA97 and UG037 trimers could be produced, although only when PGT151 or PGT145 bNAb positive‐selection columns, respectively, were used as the purification method.^58^

The HR1‐redesign concept described above was also tested in the context of the BG505 NFL‐trimer construct, as were additional variations to the length and identity of flexible linker used to replace the REKR cleavage site.^128^ Using a short, 8‐residue linker was clearly problematic, in that trimer yields were reduced (CST trimers); a short SGS linker was also unsatisfactory, with a subset of the resulting CSF trimers having a markedly atypical conformation that was not present when an SOS bond was also included (CSF‐SOS trimers). As previously reported,^130^, ^132^ using longer GGGGS linkers (10 residues) gave better outcomes, particularly when the SOS bond was also included.^128^ The resulting CSL‐SOS trimers now had a broad morphological resemblance to the products of the SOSIP and NFL or SC trimer designs. Antigenically, the resulting CFL‐SOS trimers, now designated ‘UFO’ (Fig. 1), have a slightly reduced exposure of some non‐NAb epitopes, but at the price of modest impairments to some bNAb epitopes at the trimer apex and the gp120‐gp41 interface.^128^ Overall, the extent to which the HR1 redesign improves the linker‐stabilized uncleaved trimers of the NFL/SC design seems minor, and is likely to be genotype‐dependent. In most cases, the SOS bond still appears to be highly beneficial for stabilizing the resulting trimers.

How useful these alternative designs will prove to be remains to be determined. The limited amount of published information suggests that the yields of fully purified SC‐, NFL‐, or UFO‐gp140s are comparable to that of SOSIP.664 trimers of the same genotype in transient transfection systems (i.e. 1–3 mg/L).^114^, ^128^ There are no reports of stable cell lines expressing these next generation uncleaved gp140s with which to compare the various SOSIP.664 or SOSIP.v4.1 CHO cell lines. Under standard laboratory conditions, purified trimer yields from these CHO lines are approximately 5–15 mg/L, depending on the design and genotype, and a CHO line produces approximately 30 mg/L of purified BG505 SOSIP.664 trimers under clinical grade conditions.^106^ Thus, no obvious ‘Furin penalty’ to the production of cleaved, native‐like SOSIP.664 trimers has been identified. It is also not clear that the SC‐ and NFL‐gp140 designs, with or without the additional HR1 redesign, increase the range of genotypes from which native‐like trimers can be produced; the genetic limitations on trimer formation and stability are not restricted to the C5 residues in gp120 that are most relevant to the various designs. Perhaps the most likely utilization of the SC‐, NFL‐, or UFO‐gp140s might be in the context of DNA, mRNA, or viral vector delivery systems. There is no information on the extent to which any gp140 design forms fully native‐like trimers under in vivo conditions after genetic or vector delivery; but an argument can certainly be made in favor of the regulatory simplicity of omitting the need for Furin, which is a host‐cell protein. Comparative immunogenicity studies based around the assessment of vector delivery systems that incorporate different trimer designs would be needed to address these points.

Other trimer‐design strategies are based on expressing full‐length Env proteins on the surface of stably transfected cells, followed by the detergent extraction and purification of the trimers.^89^, ^142^ The rationale is that the most truly native form of Env is the one presented on the surface of virus particles, and by extrapolation on Env‐transfected cells. Thus, the presence of the transmembrane and cytoplasmic domains has long been known to influence the antigenicity of the external domains,^143^ and, hence, may also affect their immunogenicity. As yet, very little information has been reported on the practical aspects of this approach. Several challenges that will need to be addressed include optimizing the yield, the biochemical purity, and the conformational homogeneity and integrity of the purified trimers. To increase the yield, in one approach the cleavage site between gp120 and gp41 has, again, been knocked out by mutagenesis to prevent gp120 dissociation and the consequent loss of trimer product.^142^ However, Env‐transfected cells, or virus‐like particles (VLPs) derived from them, express a range of native‐like and non‐native Env forms on their surfaces, particularly when the cleavage site is knocked out.^22^, ^144^, ^145^ In addition, partially processed and hence arguably non‐native intracellular Env proteins may be present in whole‐cell detergent extracts. A conformationally selective bNAb may be a useful part of the purification strategy, to isolate only the native Env sub‐population. The quaternary epitope‐specific PGT151 bNAb has already been shown to be valuable in this regard.^27^, ^89^ Whether this approach would be practical depends on the relative importance placed on conformational integrity versus total yield.

A conceptually related method involves VLPs. Here, the full‐length Env proteins are expressed on the surface of particles released from Env‐transfected cells, as opposed to being solubilized from the cells. We discuss the VLP method below in the ‘Particulate presentation’ section.

## 9 Immunogenicity of native‐like trimers

**Figure 3:**
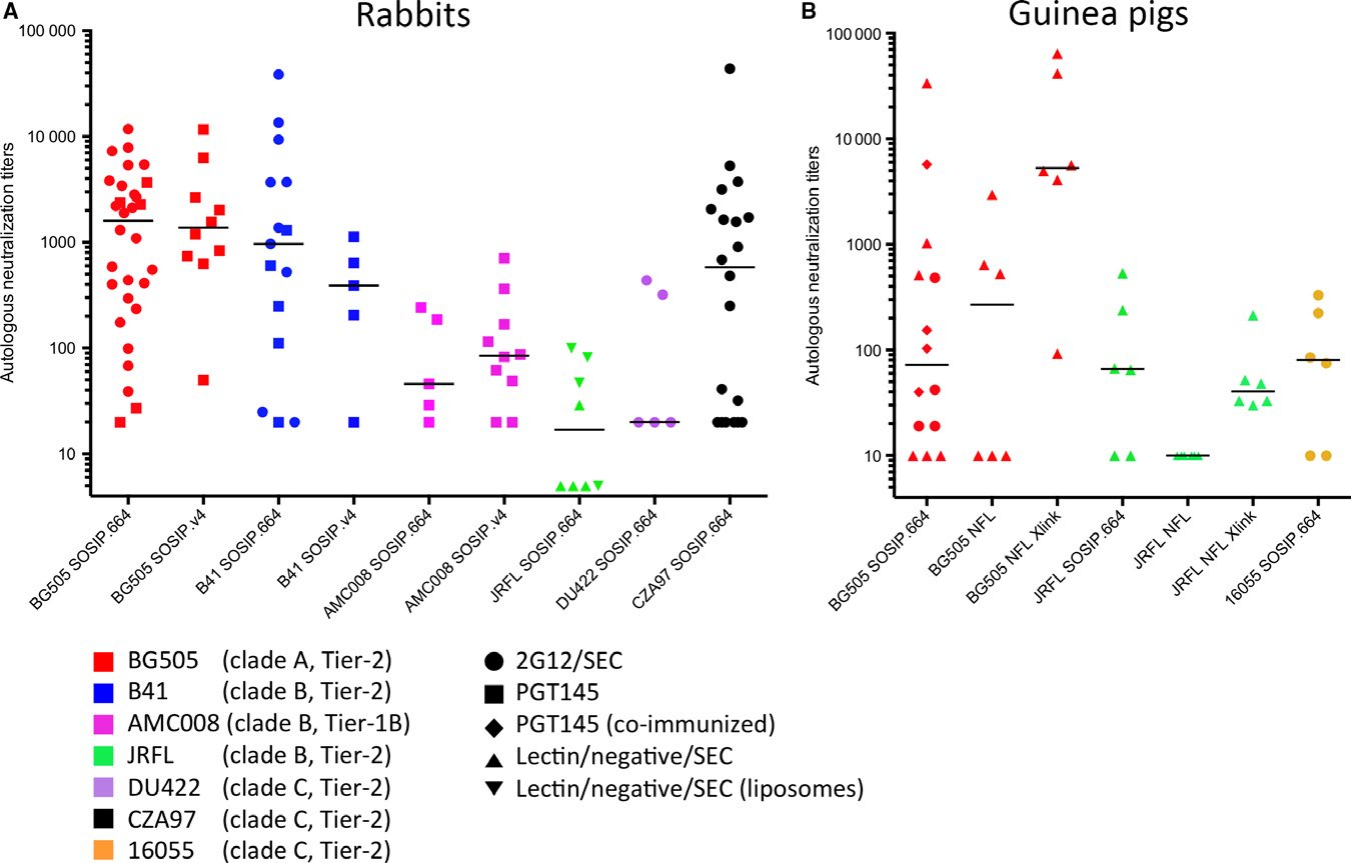
Autologous NAbs induced in rabbits and guinea pigs by native‐like trimers of different designs and genotypes and purified by different methods. The figure depicts autologous NAb titers derived from published studies that have evaluated the immunogenicity of various designs of native‐like trimers in (A) rabbits or (B) guinea pigs. (A) The rabbit data are reported in the following papers: BG505 SOSIP.664 (red)^84^, ^123^, ^150^; BG505 SOSIP.v4.1 (red)^123^; B41 SOSIP.664 (blue)^84^, ^123^, ^150^; B41 SOSIP.v4.1 (blue)^123^; AMC008 SOSIP.664 (magenta)^123^; AMC008 SOSIP.v4.1 (magenta)^123^; JR‐FL SOSIP.664 (green)^155^; DU422 SOSIP.664 (purple)^150^; CZA97 SOSIP.664 (black).^150^ The JR‐FL SOSIP.664 trimers were used as single trimers (triangles) or presented on liposomes (inverted triangles).^155^ The DU422 and CZA97 data were derived when these trimers were used as boosting reagents in rabbits previously immunized with BG505 or B41 trimers.^150^ Their immunogenicity as de novo immunogens has not yet been reported. (B) The guinea pig data are reported in the following papers: BG505 SOSIP.664 (red)^131^, ^154^; BG505 NFL (red)^131^; BG505 NFL Xlink (red)^131^; JR‐FL SOSIP.664 (green)^131^; JR‐FL NFL (green)^131^; JR‐FL NFL Xlink (green)^131^; 16055 SOSIP.664 (orange).^131^ ‘XLink’ refers to glutaraldehyde cross‐linking of trimers prior to immunization.^131^ Four guinea pigs received BG505 SOSIP.664 trimers as complexes with the PGT145 bNAb (red diamonds).^154^ On both panels, the symbol shapes indicate the affinity column‐based method used to purify the trimers: 2G12 followed by SEC (circles); PGT145 (squares); lectin followed by non‐NAb depletion and then SEC (triangles, upright for soluble trimers, inverted for liposome presentation). All the NAb titers were derived using the TZM‐bl cell assay, but not all in the same laboratory. Our experience is that titers can vary by up to threefold when nominally the same TZM‐bl cell assay is carried out with the same sera and test viruses in different laboratories. Immunization conditions, including adjuvants, also vary between different research groups and sometimes between experiments, introducing another variable of unknown magnitude. For details, the primary papers must be consulted. In addition, the genotype affects both the immunogenicity of the trimer and the overall neutralization sensitivity of the autologous virus. All the virus genotypes referred to here have Tier‐2 neutralization resistance phenotypes except for AMC008 (Tier‐1B), but there is a spectrum of sensitivities within the Tier‐2 classification. Overall, the multiple variables across the totality of experiments mean that this figure should be used only as a guide to the relative immunogenicity of different trimers of various designs and genotypes. Only directly comparative experiments, whether completed or for the future, allow more precise conclusions to be drawn

Only a few studies on the immunogenicity of native‐like trimers in animal models have been published (summarized in Fig. 3). In general, they have shown that the trimers elicit NAbs against the autologous Tier‐2 virus, as well as heterologous Tier‐1 NAbs, but without inducing the type of broadly neutralizing response to Tier‐2 viruses that is likely to be required in a successful vaccine. The induction of an autologous Tier‐2 NAb response is clearly not sufficient for a useful vaccine, but it may be a necessary first step in the creation of one; during HIV‐1 infection, bNAbs emerge from an initial autologous response over time.^2^, ^29^, ^146^, ^147^, ^148^, ^149^ Our collective task is to devise ways to mimic or improve on that process, in a way that can be translated into a practical vaccine.

In the initial report on native‐like trimer immunogens, BG505 SOSIP.664 trimers were studied in rabbits and, to a lesser extent, macaques.^84^ There was a strong and consistent NAb response to the autologous BG505.T332N virus, with titers that could exceed 1000 in some rabbits (median = 570; Fig. 3A). These NAbs were not directed to V3 epitopes, but their targets were not well defined. NAbs to heterologous Tier‐1 viruses were also elicited, and shown to be predominantly against V3 epitopes. The autologous Tier‐2 and the heterologous Tier‐1 NAb responses were completely non‐correlated. A less consistent and weaker autologous Tier‐2 NAb response to the same BG505 trimers was seen in immunized macaques. A second SOSIP.664 trimer, based on the B41 clade B genotype, also induced a strong and consistent autologous Tier‐2 NAb response in rabbits^84^, ^123^, ^150^ (Fig. 3A). In contrast to the responses to the native‐like trimers, an uncleaved BG505 pseudotrimer was very poorly immunogenic in rabbits, failing to induce any detectable autologous NAbs.^84^ The latter finding was consistent with earlier studies of various other pseudotrimers of different genotypes in small animals.^66^, ^151^, ^152^, ^153^

The BG505 SOSIP.664 trimers were also tested in guinea pigs both alone and as complexes with the PGT145 bNAb, again leading to the elicitation of autologous Tier‐2 NAbs, together with NAbs to heterologous Tier‐1 but not Tier‐2 viruses^154^ (Fig. 3B). The rationale for the formation of a complex with PGT145 was that the bNAb would lock the trimer in the closed prefusion conformation and reduce exposure of the V3. The strategy was successful as the protein–bNAb complex was more thermostable than the free trimer, and the immunogenicity of the V3 was reduced by 100‐fold. However, there was no significantly increase in the magnitude or breadth of Tier‐2 virus neutralization. An additional study of BG505 SOSIP.664 trimers in guinea pigs is summarized below.^131^

In contrast to what was seen in rabbits, macaques, and guinea pigs, no autologous Tier‐2 NAbs were induced when several sub‐species of mice were immunized with BG505 SOSIP.664 trimers.^111^ Binding antibodies and heterologous Tier‐1 NAbs were elicited, establishing that the trimers were immunogenic. Why the mice appear to be non‐responsive only at the autologous Tier‐2 NAb level remains uncertain. Among possibilities are a greater sensitivity to the possible immune‐interfering effects of Tier‐1 or non‐NAbs, compared to other species; or an inability of the mouse antibody germline repertoire to raise antibodies with the characteristics needed to penetrate the glycan shield and bind to the relevant epitopes (see below).

As noted above, there is a strong argument for stabilizing the closed, prefusion form of the trimer and minimizing its ability to sample the fully open, CD4 receptor‐bound form.^125^, ^126^ Such conformational flexibility may hinder the induction of bNAbs. Accordingly, the SOSIP.v4.1 and v4.2 trimers were designed to reduce the presentation of V3, CD4i and non‐neutralizing CD4bs epitopes without compromising the presentation of bNAb epitopes.^123^ When tested in rabbits, SOSIP.v4 trimers based on the BG505, AMC008, and B41 *env* genes induced similar autologous Tier‐1B (AMC008) or Tier‐2 (BG505 and B41) NAb titers to those elicited by the corresponding SOSIP.664 trimers (Fig. 3A). However, lower levels of V3‐directed NAbs against Tier‐1A viruses were induced. There was no increase in NAb breadth. A reduction in V3 immunogenicity was also seen when BG505 SOSIP.v4 trimers were tested in mice, but again without inducing autologous Tier‐2 NAbs in that species.^123^

In an in press manuscript, we report on the immunogenicity in rabbits of SOSIP.664 trimers based on the *env* genes of four isolates: BG505 (clade A), B41 (clade B), CZA97 (clade C), and DU422 (clade C)^150^ (Fig. 3A). These trimers were delivered either simultaneously (as a mixture of clade A + B trimers) or sequentially during prolonged immunization regimens. Autologous, Tier‐2 NAbs were induced to both the BG505‐ and B41‐based trimers when they were used as a bivalent immunogen. The clade C trimers were tested only as boosting antigens in rabbits previously immunized with the clade A and/or clade B trimers, and under these conditions they elicited autologous Tier‐2 NAbs; here, the CZA97 trimers were the stronger and more consistent immunogen, compared to DU422^150^ (Fig. 3A). The clade C trimers could also cross‐boost pre‐existing NAb responses to the clade A and B trimers. Heterologous Tier‐2 NAb responses were seen, although only inconsistently, and their breadth was limited overall. Nonetheless, cross‐neutralization of the clade A BG505.T332N virus was seen consistently in rabbits given only the clade B trimers followed by a clade C trimer boost. We describe below how the autologous NAbs induced by the BG505, B41, and CZA97 trimers are predominantly directed against specific holes in the glycan shields of the cognate viruses. The shared location of some of these holes may account for some cross‐boosting effects, and/or the cross‐neutralization of the BG505.T332N virus, although this is speculative.^150^

JR‐FL SOSIP.664 trimers, enriched for native‐like forms via a negative selection column, induced autologous Tier‐2 NAbs in rabbits and guinea pigs, but only inconsistently and weakly^131^, ^155^ (Fig. 3A, B). Overall, their immunogenicity was reminiscent of the limited responses seen in rabbits when earlier generation JR‐FL SOSIP.681 trimers were tested in rabbits, without negative selection to remove non‐native forms^156^, ^157^; and also to results seen with VLP‐associated JR‐FL trimers in various animal species (see below).^158^, ^159^

One inference from the above experiments is that JR‐FL is not a good genotype on which to base trimers aimed at inducing, initially, an autologous NAb response, perhaps because trimers based on it seem to be rather unstable.^131^ However, we are now becoming increasingly aware from that the genotype of appropriately stable native‐like trimers has a substantial influence on their immunogenicity, for reasons that will need to be understood. In our experience, multiple SOSIP trimers based on various clades and genotypes induce high (titers >100) and consistent (the majority of animals) autologous Tier‐2 NAb responses in rabbits, but a few comparably native‐like and stable trimers do not. At present, we have only provisional insights into what sequence/structure factors most influence immunogenicity, and it is possible that different factors may be involved in the induction of autologous versus heterologous Tier‐2 NAbs. One observation is that the capability of native‐like SOSIP trimers to induce autologous Tier‐2 NAbs is associated with the presence of holes in the glycan shield (see also below). Thus, trimers based on virus isolates with no glycan holes are less efficient at inducing such NAbs compared to ones that do have such holes.

Another critical variable is the animal species. Few comparative studies have been performed, but it is clear that BG505 SOSIP.664 trimers are less immunogenic (for autologous NAbs) in macaques than in rabbits and guinea pigs and that these antibodies are not induced at all in conventional mice.^84^, ^111^, ^123^, ^150^, ^154^ We assume that these observations are not likely to be unique to the BG505 genotype, implying that they are rooted in species‐dependent variations in host immunity. As the goal, of course of all native‐like trimer development programs, is to induce relevant immune responses in humans, much important information remains to be learned. In addition, all antibody titers elicited against native‐like trimers to date have the short half‐lives (i.e. a few weeks) that appear to be characteristic of anti‐Env immunogen antibody responses in general.^84^, ^160^, ^161^, ^162^ Immunology‐based solutions to these long‐standing problems also need to be sought and found.^163^

The immunogenicities of various improved SOSIP trimer designs (except for SOSIP.v4, see above) have not yet been reported, which is also true of the SC and UFO uncleaved native‐like trimers. In a recent report, the JR‐FL NFL trimer induced no autologous Tier‐2 NAbs in guinea pigs, whereas the JR‐FL SOSIP.664 trimer did so, albeit inconsistently.^131^ In the same guinea pig study, the immunogenicities of the BG505 NFL and SOSIP.664 trimers were broadly similar (Fig. 3B). However, the Tier‐2 autologous NAb response to the BG505 SOSIP.664 trimers was weaker and less consistent than seen in rabbit experiments.^84^, ^123^, ^150^ It is unknown whether the explanation is based in species‐dependent immunogenicity variations or in how what are nominally the same BG505 SOSIP.664 trimers were purified: lectin‐chromatography followed by a non‐NAb depletion column,^131^ a 2G12 affinity column followed by SEC^84^, ^150^, or a PGT145 affinity column.^123^ Glutaraldehyde cross‐linking improved the ability of BG505 NFL trimers to induce autologous Tier‐2 NAbs in guinea pigs. Cross‐linking also improved the immunogenicity of JR‐FL NFL trimers, to approximately the same level of JRFL SOSIP.664 trimers that had not been cross‐linked^131^ (Fig. 3B). A similar trimer cross‐linking method also increases the immunogenicity of BG505 and B41 SOSIP.664 trimers in rabbits (Q. Sattentau, Personal Communication).

## 10 Specificity of the NAb response to native‐like trimers

As summarized above, native‐like trimers based on multiple genotypes induce autologous Tier‐2 responses in rabbits (Fig. 3A). An obvious question is what the nature of the epitopes is that are recognized by the serum antibodies, and whether these autologous NAbs can be broadened. Initial studies showed that the autologous NAbs to the BG505 SOSIP.664 trimer were not directed at V3 epitopes, a conclusion since reached in analysis of immunization studies involving other SOSIP trimers.^84^, ^123^, ^131^, ^150^ This observation stands in marked contrast to the Tier‐1 NAbs induced by various pseudotrimers, which are depleted when V3 peptides are added to neutralization assays.^63^, ^164^ That finding is also true of the Tier‐1 NAb response to SOSIP trimers.^84^, ^123^ Of note is that the V3‐dependent Tier‐1 NAb responses to the BG505 and other native‐like trimers are not correlated with the autologous Tier‐2 responses.^84^, ^150^ Different epitope specificities are involved. Early mapping studies involving the use of soluble competitors in neutralization assays, combined with a panel of BG505 mutant viruses, implicated glycan‐influenced epitopes as the targets for the autologous NAbs, and suggested that the responses may differ among the rabbit cohort. However, it was not possible to identify the epitope(s) with greater precision.^84^ NAbs raised against free and PGT145‐complexed BG505 SOSIP.664 trimers in guinea pigs also neutralized the autologous virus, but the maternal MG505 virus (see below) was neutralized much less efficiently. The sera were also shown to contain antibodies that blocked trimers from binding to CD4, but the relevant epitope(s) was not identified in any more detail.^154^ More recent studies have provided a much more detailed insight into the nature of the autologous NAb response in rabbits to the BG505 trimers,^150^, ^165^ as well as those to the B41 clade B and CZA97 clade C trimers.^150^

A key observation from the Binley group underpinned the analysis of the response to native‐like trimers. A small subset of rabbits immunized with VLPs expressing native‐like JR‐FL trimers developed autologous Tier‐2 NAbs that were mapped to the lack of an N‐linked glycan at residue 197.^144^ Thus, a mutant JR‐FL virus in which a glycan was introduced at this position became resistant to the NAbs present in the rabbit serum. As most viruses contain a glycan at position 197 they were not neutralized by the rabbit sera, which provides a mechanistic explanation for the narrow specificity of the overall response. However, several heterologous Tier‐2 viruses became sensitive to the rabbit sera when their 197 glycans were removed.^144^ The authors extrapolated from their data to suggest that many Tier‐2 viruses would have specific, but narrow, vulnerabilities to antibodies created by ‘holes’ in their glycan shield. Thus, the loss of a glycan for whatever reason (neutralization escape in vivo, an increase in fitness, etc.) could expose the underlying protein surface as a site for antibody binding. That same area could also, by extension, be immunogenic. These predictions turned out to be accurate.

The BG505 isolate lacks glycans at positions 241 and 289 creating a hole in the glycan shield, whereas most virus isolates have glycans at these positions (Fig. 2D). This hole appears to be immunogenic on BG505 SOSIP.664 trimers in that the most common autologous NAb response to these trimers in rabbits targets this hole.^150^ More specifically, neutralization by most of the rabbit sera is reduced, and often ablated, by the introduction of glycan residues at positions 241 or 289.^150^ Modeling suggests that either glycan can impede access to the same underlying peptide surface. The nature of residue 241 is particularly important to the epitope because the S241K point substitution also creates a resistant virus mutant. However, a substantial minority of BG505 trimer‐immunized rabbits also, or instead, develops autologous NAbs that do not map to the aforementioned glycan hole. That alternative epitope(s) remains to be identified. A consistent observation among the immunized rabbits is that the sera do not neutralize clones of the MG505 virus isolated from the mother of the BG505 infant. However, there are multiple sequence changes between the MG505 and BG505 viruses, including but not limited to the presence versus the absence of a glycan at position‐241.^150^ The isolation of MAbs from other BG505 SOSIP.664 trimer‐immunized rabbits confirmed that one of the dominant NAb responses was directed against the glycan hole at position‐241.^165^

The rabbit response to the B41 SOSIP.664 trimer is consistently to a glycan hole at residue 289, and the dominant response to the CZA97 trimer is centered on the V4 region, where a glycan site is missing at position 411.^150^ Taken together, the narrow specificity of these autologous NAb responses reflects the rarity of the glycan holes across panels of test viruses, combined with additional sequence diversity within the protein surfaces at the base of the holes. It remains to be seen whether these hole‐directed responses are ‘dead‐end’ responses that cannot be broadened or whether they can be exploited to develop bNAbs. Observations that several bNAb lineages may have been initiated by Env variants with a hole in their glycan shields support the latter, more positive, perspective. Thus, the VRC01‐lineage was probably initiated by an Env trimer that lacked glycans at N276 and N460,^166^ while the lack of the N137 glycan may have been instrumental for the PGT121‐lineage of bNAbs.^114^

## 11 Particulate presentation of native‐like trimers

HIV‐1 Env vaccine subunit development strategies can be guided by the success of two protective antiviral subunit vaccines, those against hepatitis B virus (HBV) and human papillomavirus (HPV). Both vaccines present the viral surface protein in particulate form. HBV surface antigen self‐assembles into lipid‐protein particles of approximately 22 nm that display 24 copies of the S‐protein as surface protrusions,^167^, ^168^ while the L1 protein of HPV self‐assembles into protein VLPs of approximately 55 nm that typically comprise 72 L1 pentamers.^168^, ^169^ Presenting key immunogenic proteins as multivalent, particulate antigens is also proving to be a highly promising approach for influenza virus and Epstein–Barr virus (EBV) vaccines.^170^, ^171^, ^172^

The concept of a particle‐based vaccine is well founded in basic immunological principles, among them being how the mammalian immune system is set up to respond to virus‐sized antigens; the avidity advantages conferred by the proximity of multiple copies of a key antigen on the surface of a particle; and the possibility that at least some useful T‐cell‐independent antibody responses can be triggered when multiple B‐cell receptors are triggered by a regular array of immunogens, something that soluble forms of the same proteins cannot mimic. Early experiments using hapten repeats suggested that, at minimum, a particulate antigen should present 12–16 copies of a key epitope(s).^175^ Schiller and Chackerian have argued that the HIV‐1 virion carries too few Env trimers on its surface to trigger B‐cell activation efficiently, as yet another way to evade the humoral immune system.^174^ The stiffness of the antigen carrier might also affect B‐cell activation, with a rigid carrier triggering stronger B‐cell activation signals than a softer, more deformable one.^175^

The size of the particle also determines how it traffics to the draining lymph nodes, a prerequisite for an optimal B‐cell response. After vaccination, small soluble proteins rapidly disseminate into the systemic circulation and hence are poorly taken up into lymphatic sites. Conversely, very large particles are easily trapped within the tissue near the injection site, and hence may not reach the lymph nodes either. The optimal particle size appears to be 20–50 nm,^176^ the upper end of this range (50 nm) matching the average mesh size of extracellular matrix in connective tissues.^176^, ^177^ The size restrictions are apparently different for elastic (i.e. deformable) particles, such as liposomes and enveloped VLPs, than for more rigid particles that cannot change their shape when trafficking through small spaces.

Many attempts have been made to present various forms of HIV‐1 Env in particulate form, but without any notable successes from the perspective of inducing bNAbs. Initial efforts to present native‐like trimers on particles were based on enveloped VLPs that are secreted from Env‐transfected cells and then purified.^144^, ^145^, ^178^, ^179^ One advantage of the VLP design is that, as noted above, the transmembrane and/or cytoplasmic domains of Env increase the stability and conformation of the immunogenic external domains; a second is the immunological benefits of multivalency. Conversely, VLPs are not easy to make and then purify in high quantities, which creates challenges from the translational perspective. The low Env density on VLPs is another concern, although here truncating the gp41 cytoplasmic tail can be beneficial.^174^, ^180^ Env proteins presented on VLPs (and, by extension, on the Env‐transfected producer cells) are not homogeneous, with both native‐like and non‐native conformations detectable in antigenicity assays.^22^ A clever solution to this problem was to exploit the greater protease vulnerability of the open, splayed‐out gp120 subunits of non‐native Env, and thereby use these enzymes to clean up the VLP surface by digesting away the latter, unwanted Env forms. Using this method, VLPs can be made that carry exclusively native‐like trimers on their surface, provided the inter‐subunit SOS bond is engineered into the construct to confer the required stability and overcome gp120 dissociation.^144^, ^145^ The much greater protease sensitivity of the non‐native Env forms on VLPs (and by extension the producer cells), compared to the more compact, native‐like trimers, mirrors what was seen when soluble uncleaved pseudotrimers and SOSIP.664 trimers were similarly compared.^58^, ^71^, ^79^ Overall, the instability issues associated with incomplete cleavage that we summarized earlier probably have qualitatively similar consequences in the two contexts but may be less profound quantitatively with full‐length Env.

Tested in animals, the protease‐treated JR‐FL SOS Env VLPs were able to induce sporadic autologous Tier‐2 NAb responses that, for some sera, could be mapped to specific holes in the glycan shield.^158^, ^159^ The poor immunogenicity of JR‐FL‐based soluble, native‐like trimers alluded to earlier^131^, ^155^, ^156^, ^157^ may suggest that better results could be obtained in the VLP context if a different Env genotype were used instead.

A different approach to presenting Env proteins in particulate form involves coating them onto the surface of liposomes. For example, liposomes can be made that incorporate a chemically modified lipid that serves as a recognition site for a His‐tag that is added to the C‐terminus of Env. Simply mixing the liposomes and the Env protein allows the latter to be captured non‐covalently, but stably, onto the particle surface. An initial study using an uncleaved pseudotrimer as the Env antigen showed that it was possible to purify enough Env‐coated liposomes for testing in animal immunogenicity experiments.^181^ Given the nature of the incorporated Env protein, only Tier‐1 NAbs and binding antibodies were induced, although there were titer increases compared to the soluble gp140 comparator group.^181^ Because His‐tags can be routinely incorporated into the design of SOSIP trimers and their uncleaved native‐like derivatives, this approach offers considerable flexibility from the perspective of the Env genotype. Thus, liposomes that display JF‐FL SOSIP.664 trimers were made by copying the above method.^155^ The trimer‐coated liposomes appear to have appropriate properties when tested in in vitro systems, including the triggering of B‐cell responses via trimer–BCR interactions. However, there was no meaningful improvement in immunogenicity over the corresponding soluble JR‐FL SOSIP trimers when the liposomes were tested in rabbits^155^ (Fig. 3A). Again, what appears to be the generally poor immunogenicity of JR‐FL Env proteins may be a factor. It seems unlikely that this initial report will be the definitive statement on the liposomal presentation of native‐like trimers, given the potential of the method and its capacity for improvement.^176^, ^181^, ^182^, ^183^

In principle, a ‘protein‐only’ presentation of a native‐like trimer might be more easily translatable at the clinical level, compared to VLPs and perhaps liposomes. Ferritin‐based constructs have shown promise in experimental vaccines against influenza virus and EBV.^170^, ^171^, ^172^In this system, 24 ferritin monomers spontaneously assembles into regular particles of approximately 12 nm in diameter. If an HIV‐1 Env protein is fused appropriately to the ferritin moiety, it is presented on the particle's external surface. In principle, eight trimers could be displayed on a ferritin particle. A ferritin‐based nanoparticle displaying multiple copies of cleaved, native‐like BG505 SOSIP.664 trimers was shown in pilot experiments to be modestly more immunogenic in mice and rabbits than the corresponding soluble trimers.^140^ The initial design is capable of improvement from several perspectives, including the homogeneity of both the particles and the trimers presented on their surface. For example, some of the BG505 SOSIP.664 trimers on ferritin particles remained uncleaved and hence were non‐native.^140^ Trimers of different genotypes or designs could be presented on different ferritin nanoparticles, or perhaps even on the same particle. The ferritin‐particle design is also compatible with the native‐like uncleaved SC‐, NFL‐ or UFO‐trimer constructs, eliminating the need for a furin‐mediated cleavage event to take place in what may be a sterically constrained environment created by the close packing of multiple trimers into a confined space. The presentation of UFO‐trimers on ferritin nanoparticles has recently been reported.^141^

As noted above, ferritin particles can present a maximum of eight trimers, which might be below the optimal number of epitope repeats (minimum of 12–16, see above). The most accessible site on ferritin‐displayed trimers is likely to be the apex region, which includes epitopes for bNAbs such as PG9/16, PGT145, PGDM1400, and others. These bNAbs bind trimers with a stoichiometry of 1 (i.e. eight total binding sites per trimer‐ferritin particle), which again may be sub‐optimal for B‐cell activation. Thus, somewhat larger particles that present more copies of the trimer may be advantageous from the B‐cell activation perspective. One such system is another self‐assembling nanoparticle based on dihydrolipoylacetyltransferase (E2p) from *Bacillus stearothermophilus*.^141^ The E2p proteins self‐assemble into 60‐mer particles with a diameter of approximately 23 nm. As with the ferritin‐based system, the trimers and the nanoparticle components are genetically fused, such that 20 trimers are presented on the surface of the assembled particle.^141^ Newer, icosahedral nanoparticle designs might also be valuable.^184^, ^185^ Other biodegradable nanoparticle approaches to Env trimer presentation could include chitosan‐ or poly(D,L‐lactic‐co‐glycolic acid)‐based particles and ones based on ‘bacterial ghosts’.^186^, ^187^, ^188^, ^189^

Most of the above particulate antigen presentation systems have the additional potential for including immunostimulatory molecules such as TLR activators on their surfaces or within their internal spaces, with the goal of further stimulating desirable immune responses.^63^, ^176^, ^184^, ^185^, ^187^, ^189^ In principle, such an adaptation could allow the targeted delivery of an adjuvant component directly to the site where an immune response to the trimer component is initiated.

## 12 Lineage vaccines based on native‐like trimers

As noted above, bNAbs are not produced early in HIV‐1 infection but can emerge in a subset of people as a result of virus–host co‐evolution events that shape the initial autologous NAb response over time. Now that we know that native‐like trimers can induce autologous NAbs, is it possible to mimic the evolutionary process toward bNAbs by designing appropriate trimer variants and a longitudinal immunization scheme? One approach, referred to as a ‘natural lineage vaccine’, is based on longitudinal *env* gene sequences from infected people who did eventually generate bNAbs.^2^, ^29^, ^146^, ^147^, ^148^, ^149^ An alternative, but not necessarily mutually exclusive, method is the ‘designed lineage vaccine’ in which the Env immunogens are specifically engineered to steer the antibody response toward a defined bNAb epitope.^190^, ^191^, ^192^, ^193^, ^194^, ^195^, ^196^, ^197^, ^198^, ^199^ Here, we will address the suitability of native‐like trimers as the basis of the overall immunization schemes. In principle, the trimer immunogens could be presented in either soluble or particulate form, and there may be strong grounds to prefer the latter approach when kick‐starting bNAb lineages. At present, however, the limited amount of available information is restricted to soluble trimers.

A *sine qua non* for a ‘natural lineage vaccine’ is a set of longitudinal *env* gene sequences from an infected individual who developed bNAbs. That sequence set can then be used to engineer the corresponding native‐like trimers of the SOSIP or alternative designs. Here, the accumulating knowledge of the sequence requirements for forming native‐like trimers and/or how to purify them is invaluable. Thus, an attractive set of longitudinal *env* sequences is only valuable in practice if each of the key variants can be used to make fully native‐like trimers. It must be taken into account that variations that accumulate in the *env* gene over time may compromise trimer quality. In other words, virus escape from neutralization may come at a price, the cost being a reduction in fitness that is attributable to a somewhat impaired trimer. Given the additional fragility of soluble trimers, even SOSIP‐stabilized ones, compared to their full‐length, membrane‐associated counterparts, the fitness factor can and does loom large in the design process. Moreover, *env* clonal variation may generate sequence variants that could seem innocuous at the virus level, but do have a phenotype in the context of a soluble trimer. We have encountered these scenarios when working on SOSIP trimers based on *env* sequences derived from the BG505 virus‐infected infant during the time when bNAbs emerged. Thus, some of the trimers based on later sequences were markedly more heterogeneous and less native‐like than the BG505 SOSIP.664 prototype that is based on the week‐6 founder sequence. Design tweaks based on knowledge of the determinants of trimer formation combined with appropriate purification systems have, however, enabled us to produce a longitudinal set of BG505‐based native‐like trimers for testing, initially, in animals. Other ‘natural lineage’ trimers we are pursuing as immunogens are derived from the Amsterdam Cohort, which includes many examples of infected people who generated bNAbs. From among them, we selected ‘Elite Neutralizers’ whose bNAbs were at the top of the spectrum for breadth and potency, and are creating lineage trimers based on longitudinal sequences.^34^, ^200^

In the ‘designed lineage’ approach, Env proteins are specifically engineered to kick‐start a specific bNAb development pathway. This process involves engaging the BCRs on naive B cells that express the unmutated precursor of a bNAb, the germline (gl)‐antibody. It has become clear in recent years that recombinant Env glycoproteins based on ‘standard’ HIV‐1 sequences bind the gl‐versions of bNAbs inefficiently, if at all.^191^, ^203^, ^204^, ^205^ Arguably, if HIV‐1 Env proteins were widely seen by the human gl‐antibody repertoire, the virus would never have become established as a pandemic pathogen. We must, therefore, redesign the Env proteins to enable them to engage the gl‐bNAb precursor B cells. Critical tools here are recombinant antibodies based on the inferred sequences from the human germline.^147^, ^191^, ^203^, ^204^, ^205^, ^206^ Although these antibodies are referred to as gl‐bNAbs, they generally do not neutralize HIV‐1 efficiently and they react poorly with Env proteins including native‐like trimers.^191^, ^195^, ^201^, ^202^, ^203^ Their value is that they serve as a template for structure‐guided design efforts that are intended to increase the affinity of Env binding; and as a device to measure the outcome of the engineering efforts. Most of the work in this area to date has been carried out using constructs based on gp120 monomers such as the engineered outer domain (eOD) or the 426c gp120‐core families of proteins.^192^, ^193^, ^194^, ^196^, ^197^, ^198^ The design of these constructs generally involves deleting variable loops and/or glycan sites to increase the accessibility of the CD4bs, which is consistent with their targeted focus on the CD4bs family of bNAbs.^192^, ^193^, ^194^, ^196^, ^197^, ^198^

We see four principal reasons why native‐like trimers are an appropriate platform for designing gl‐targeting immunogens. First, several bNAb epitopes are only present on the trimer, that is, those directed against quaternary structure‐dependent epitopes at the trimer apex and the gp120‐gp41 interface.^26^, ^27^, ^28^, ^30^, ^31^, ^32^, ^33^, ^34^, ^81^, ^118^ Considering the nature of these epitopes, it is unlikely that the gl‐precursors of those bNAbs will be reactive with simpler forms of Env protein. Second, some ‘standard’ SOSIP trimers can bind some gl‐bNAbs despite not being specifically engineered to have this property, which provides a solid baseline for further improvement.^195^, ^205^, ^206^ Third, although bNAbs against the CD4bs are reactive with multiple forms of Env, constraints on their angles of approach that apply only to the native trimer and not gp120 monomers or gp140 pseudotrimers are an important influence on the binding interaction.^80^ In other words, monomer or pseudotrimer immunogens elicit antibodies that approach the CD4bs from an inappropriate angle, bind poorly if at all to the trimer, and hence are non‐neutralizing for Tier‐2 viruses.^80^ Given that the angle of approach does not appear to change drastically during maturation from gl‐Ab to bNAb,^114^, ^193^, ^207^, ^208^, ^209^ it is unlikely that the initial antibodies can be guided to mature into bNAbs. It is even possible that the initial triggering of such ‘dead‐end’ pathways may interfere with the induction of CD4bs Ab specificities that do have appropriate approach angles. Finally, trimers may be able to induce the gl‐precursors of multiple bNAb epitope clusters, not just ones associated with the CD4bs.

Accordingly, our group used structural and other insights to engineer germline‐targeting (GT) SOSIP trimers so that they would engage multiple gl‐bNAbs in vitro and, by extension, in vivo. The most advanced of the new trimers is BG505 SOSIP.v4.1‐GT1, which was based on the stabilized SOSIP.v4.1 construct described above.^123^ While SOSIP.664 and SOSIP.v4.1 trimers bind multiple bNAbs in vitro, their reactivity with gl‐precursors is more limited.^195^ Thus, the BG505 SOSIP.664 trimer does bind (with moderate affinity) to the PG9/16, CH01, and 3BC315 gl‐bNAbs in vitro, but it fails to react with several others including the precursors of all the CD4bs bNAbs tested to date.^195^ The SOSIP.v4.1‐GT1 trimer was therefore designed by removing steric clashes that hinder the binding of various gl‐bNAbs, and by creating favorable new antibody–antigen contacts that promote their binding (Medina‐Ramirez et al., unpublished data). The new trimer now bound efficiently to the VRC01, PGV19, and 12A12 CD4bs gl‐bNAbs and weakly to the CH103 gl‐precursor that targets the same cluster. In addition, its affinity for three trimer‐apex gl‐bNAbs (PG9, PG16, CH01) was improved. A 3.2 Å crystal structure of the SOSIP.v4.1‐GT1 trimer, when compared to its SOSIP.664 precursor, facilitated a mechanistic understanding of its improved engagement of gl‐bNAbs (Medina‐Ramirez et al., unpublished data). When tested in knock‐in mice expressing the gl‐precursors for VRC01 (CD4bs), the SOSIP.v4.1‐GT1 trimer induced serum antibodies of the appropriate specificity (Medina‐Ramirez et al., unpublished data).

Although of considerable value for immunogen testing and screening, the various gl‐bNAb knock‐in mouse models do have substantial limitations and are considered to be a ‘low‐bar’ compared to wildtype animals, particularly humans. Trimers with much higher affinities for germline antibodies will probably be needed in humans, because the frequency of the B‐cell targets is very much lower than in the engineered mice.^194^, ^196^ In addition, the knock‐in mouse models do not predict how well the same immunogens would perform in a more complex environment where naive B cells with different affinities and specificities compete for the available antigen supply.^210^, ^211^ Mechanisms such as epitope immunodominance and subdominance may also be highly relevant in humans; specifically, any immunodominant non‐NAbs that are induced may impede the induction or maturation of the desired bNAbs.^211^ Here, ‘easy’ targets for the antibody response, such as the peptidic V3 non‐NAb epitopes, may be problematic.^123^, ^211^

Like its precursors, the BG505 SOSIP.v4.1‐GT1 trimer binds mature CD4bs bNAbs, such as VRC01 with high affinity, but not the F105 and b6 CD4bs antibodies that do not neutralize Tier‐2 viruses. Thus, the trimer has an appropriately constrained CD4bs region, with the desired epitopes available and the unwanted ones sterically occluded. This property implies that SOSIP.v4.1‐GT1 may have the selectivity to induce VRC01‐like bNAbs without activating ‘off‐target’ lineages for non‐NAbs like F105 and b6 in vivo. As noted above, the unchanging angle of approach of Abs to the trimer during the evolution of bNAbs^114^, ^193^, ^207^, ^208^, ^209^ implies that it may not be possible to ‘rescue’ off‐target lineages by further boosting. In summary, the SOSIP.v4.1‐GT1 trimers may be able to specifically shape the selection and development of CD4bs bNAb lineages.

In contrast, the 426c and eOD designs of gl‐targeting Env immunogens, intended to induce gl‐precursors of the VRC01‐family, lack the trimerization‐induced constraints on the CD4bs and hence also present unwanted non‐NAb epitopes. Whether and to what extent this design feature matters for the induction of VRC01 gl‐bNAbs remain to be determined experimentally. Thus, one of the prime‐boost immunization strategies we are now pursuing involves using a 426c protein to activate Abs with multiple approach angles to the CD4bs, then boosting with the SOSIP.v4.1‐GT1 trimer to select for ones with approach angles that are compatible with trimer binding (and, by extrapolation, Tier‐2 neutralization).

‘Off‐target’ specificities may account for why the eOD‐GT6 construct, which is based on gp120‐OD monomers, induced only non‐NAbs in gl‐VRC01 knock‐in mice.^194^ When two gl‐targeting immunogens (eOD‐GT6 and 426c.TM_4_ΔV1‐3) were tested in knock‐in mice bearing the mature or germline versions of the 3BNC60 bNAb heavy chain, both could engage germline heavy chain B cells and drive the appropriate selection and subsequent affinity maturation of the mouse light chain.^196^ In contrast, BG505 SOSIP.664 trimers lacked this property, probably because the N276 glycan generally obstructs access to the 3BNC60 epitope on native‐like trimers.^196^ However, when given to mature heavy chain knock‐in mice, the same SOSIP.664 trimers selected a highly restricted set of mouse light chains and induced 3BNC60‐like bNAbs. The gl‐targeting eOD‐GT6 protein was much less effective in this regard, presumably because the lack of constraints on the angle at which antibodies can approach the CD4bs on this immunogen allowed the recruitment of a more diverse set of light chains and thereby favored the induction of non‐NAbs over bNAbs.^196^ All Env immunogen strategies that seek to induce gl‐bNAbs of the VRC01 class, be they based on native‐like trimer, eOD or 426c designs, face the same problem of how to overcome the blocking effect of the N276 glycan that has evolved to shield the conserved regions associated with the CD4bs.

Because the BG505 SOSIP.v4.1‐GT1 trimer binds multiple gl‐bNAbs to several epitope clusters, and not just the CD4bs, it may be able to target a greater proportion of the human gl‐antibody repertoire and increase the chances of activating at least one family of gl‐bNAb B cells. The current GT1 trimer can be further improved by structure‐guided design changes, and we have already created variants with a markedly improved affinity for several gl‐bNAbs. In addition, we have applied the same methodology to make broadly similar GT1 trimers based on other genotypes. The presentation of gl‐targeting trimers on nanoparticles may also provide an avidity boost that increases the chances of an encounter with gl‐bNAb B cells that are much less frequent in humans than in knock‐in mice. Whether it is preferable to try to design a ‘universal germline‐targeting trimer’ or a set of different trimers that each targets a specific gl‐bNAb cluster is another question to settle experimentally. Whatever the outcome, any induced gl‐bNAbs would need to be carefully shaped by appropriately designed boosting trimers that drive the maturing response toward antibodies with properties resembling bNAbs. This task will inevitably be challenging, irrespective of the design of the gl‐targeting priming immunogen.

## 13 Conclusion

The identification of ways to make and purify authentically native‐like trimers based on multiple Env genotypes has provided a fresh impetus to immunization design programs aimed at the induction of bNAbs. The regular discovery of new bNAbs, including ones to previously unrecognized epitopes, and the increasingly detailed information on the structure of trimers (alone and as bNAb complexes), together provide access to a set of tools for further immunogen design improvements that was unavailable only a few years ago. In addition, we have collectively gained an ever‐increasing understanding of the immunology of bNAb emergence and evolution, providing additional guidance about how to accomplish our goals. HIV‐1 has evolved formidable defenses against humoral immunity. Overcoming them requires that we meld these several scientific sub‐strands into a coordinated strategy involving the optimal formulation and presentation of specifically designed Env proteins. It seems likely that native‐like trimers will be at least part of any solution that may eventually be devised to the long‐standing HIV‐1 vaccine problem.

## Conflict of interest

R. W. S. and J. P. M. are listed on patents concerning stabilized Env trimers.
